# Investigation of natural environmental radioactivity concentration in soil of coastaline area of Ado-Odo/Ota Nigeria and its radiological implications

**DOI:** 10.1038/s41598-019-40884-0

**Published:** 2019-03-12

**Authors:** E. S. Joel, O. Maxwell, O. O. Adewoyin, O. C. Olawole, T. E. Arijaje, Z. Embong, M. A. Saeed

**Affiliations:** 1 Department of Physics, Covenant University Ota, Ogun State, Nigeria; 2 Faculty of Applied Science and Teknologi, (FAST) Universiti Tun Hussein Onn, Malaysia Pagoh Campus. Km 1, Jalan Panchor, 84600 Muar, Johor Malaysia; 3 Division of Science and Technology, University of Education Township, Lahore, Pakistan

## Abstract

Natural radioactivity in coastaline area soil of Ado-Odo/Ota has been carried out to ascertain the presence of radionuclides using gamma-ray spectroscopy (HPGe detector). The result showed that U-238, Th-232 and K-40 ranged from 24 ± 7–49 ± 10; 67 ± 6–120 ± 9 and 88 ± 17–139 ± 20 Bqkg^−1^ respectively. The radium equivalent for the samples ranged from 132.51 to 230.91 Bqkg^−1^ with mean value of 185.89 Bqkg^−1^. The mean value for the gamma dose rate for the soil samples was estimated to be 81.32 nGyh^−1^. The estimated values of annual effective dose equivalent ranged from 0.61 to 1.07 mSv y^−1^. The estimation of alpha index representative (Iα) ranged from 0.12 to 0.24 with mean value of 0.21 while the gamma representative index ranged between 0.465 and 0.810. The activity utilization index of the soil samples ranged from 1.09 to 1.89 with mean value of 1.53. The radiological implication in the study area has shown that the soil samples with gamma dose rate value of 89.99 nGyh^−1^, 94.39 nGyh^−1^, 97.40 nGyh^−1^ and 101.04 nGyh^−1^ respectively are higher than the recommended value of 80 nGyh^−1^ and may pose health implication for long term exposure.

## Introduction

Natural radiation is embedded in environment and humans are continuously being exposed to it due to every day interaction with the environment just as the heat rays from the sun and light^1,2^. For instance, cosmic radiations are part of occurrence of background radiation, however it has tendency to tunnel through the atmosphere from outerspace, immerse us in a constant flux of radiation. The existence of low-level radiation that emanates from soil or rock is as a result of isotopes of their radioactive elements that are part of rocks and minerals in the Earth’s crust^1,3^. Soils are produced as a result of wearing away of these minerals and rocks and consist of radio-active elements. The estimation of natural background emission in which Man is exposed to annually has been rated to be 1.1 mSv. Sources of this radiation comes from the rays of cosmic body (0.35 mSv), air (0.05 mSv) etc^1^. In terms of average, 67.6% of natural background radiation has been accounted for the exposure of individual compared to others such as occupational exposure, releases from nuclear industry and medical radiation fall out etc. The study have shown that basement complex rocks such as igneous and metamorphic rocks are associated with high radioactive radiation while low radiation has been associated with sedimentary rock^4,5^. Of importance in the study of radionuclides are U-238, Th-232 and K-40 due to the dangers these radionuclides poses in terms of silent environmental hazard impacts especially on human (mostly health related issues). The research on radio-nuclides in rocks and soils has been on the increase across the globe (both at the individual level and organizations) in the last ten to twenty decades due to the health risk that it might pose on the populace or individual as a result of anthropogenic activities on it^6–12^. Due to the variations in geological formation that exist on the solid earth upon which man stands for his sustainance, the need to ascertain the radioactive concentration level in the soil samples of such formation cannot be underestimated for the purpose of evaluating the possible radiological effects on the people that resides within the such geological formation considering the population of people residing as a community in such formation. Therefore the aim of this study is to evaluate natural environmental radioactivity concentration present in coastaline area soil formation of Ado-Odo/Ota and its possible radiological effects on the residents.

## Study area and its geological settings

**Figure 1:**
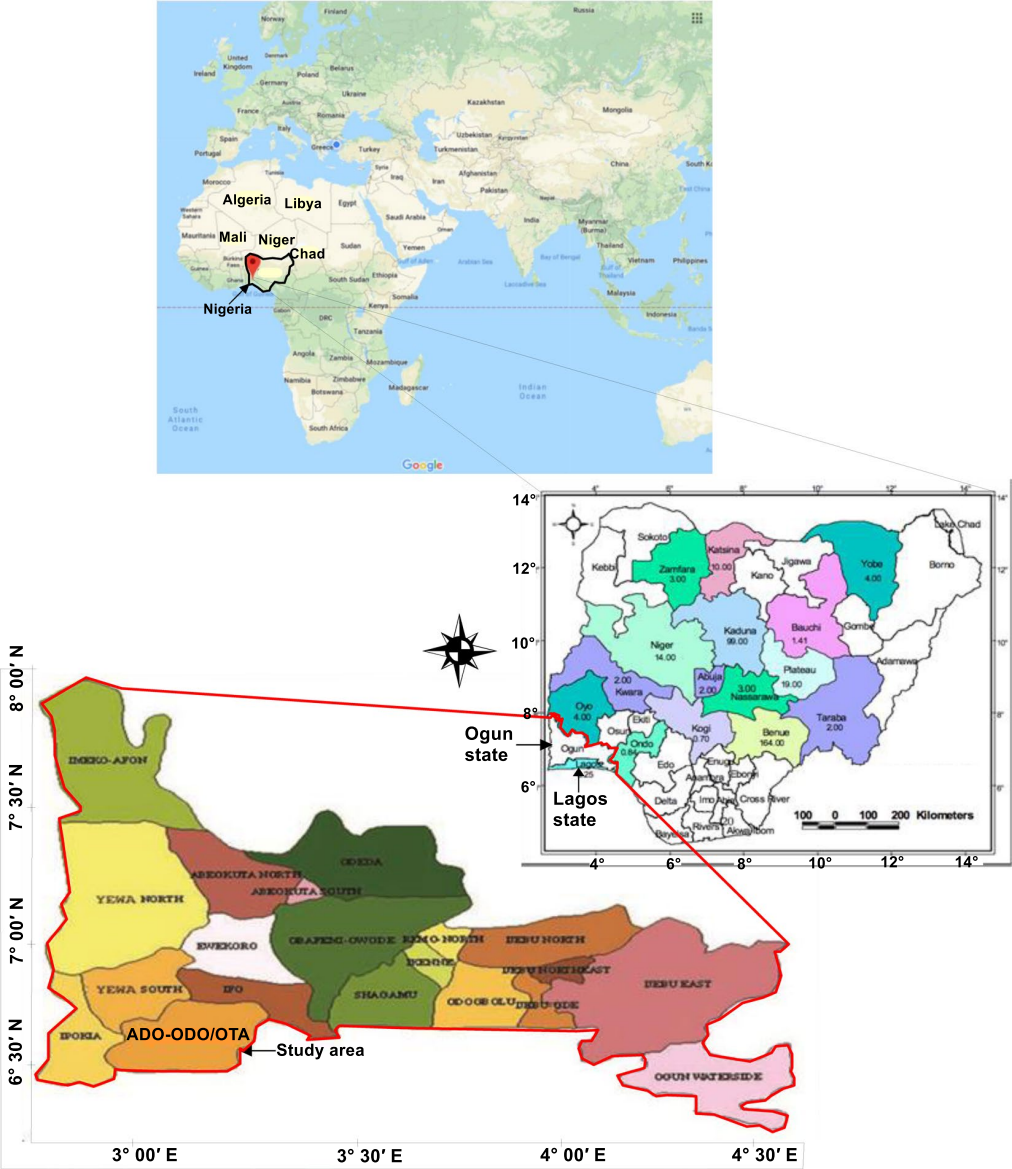
Map data© 2018 google.

The Ado-Odo/Ota area (Fig. 1) lies between longitude 3°0′00″–3°15′00″E and latitude 6°30′00″–7°00′00″N within the Dahomey basin which is one of the sedimentary terrains of Nigeria and is located at the southern part of Ogun State sharing boundary with Lagos State and it is characterized by lowlands, valleys and hills (undulating terrain). Generally, Arabian and African continent are made up of a PreCambrian basement of crystalline meta-sedimentary, igneous and meta-igneous rocks. This crystalline basement is overlain by series of geological settings ranging from volcanic and sedimentary sequences to unconsolidated Cenozoic sediments^13^. Nigeria is on the Pan-African mobile belt, which separates Congo Cratons and West Africa. The two major geological formations that spread in equal proportion are the sedimentary Basins (Upper Cretaceous in age) and Basement rocks (Pre-Cambrian in age)^14^. The local geology of the study area is made up of sedimentary rock sequence of Dahomey Basin which extends from the eastern part of Ghana through Togo and Benin Republic to the western margin of the Niger Delta. The geological succession in the basin is as follows: the structure of the basin is a simple monocline against the basement which underlies the sedimentary rocks at varying depths. The dips are reportedly 1° or less to the south and southwest^15^. The sequence of the rocks underlying the study area is as follows: Abeokuta Formation which is Cretaceous in age (Senonia) and this formation lies conformably on the Basement Complex in the north and Ewekoro Formation in the north-east. It is the oldest sedimentary formation, having thickness of 250–300 m^15^. It consists of arkosic sandstones and grits, tending to be carbonaceous towards the base^15^. Overlain the Abeokuta Formation is Ewekoro/Oshosun/Akinbo Formation which is Paleocene. This consists of a series of sandstone, shales, limestones and clays varying between 100 to 300 m in thickness. This formation is followed by Ilaro Formation (Tertiary age - Eocene). It consists of fine to coarse sands alternating with shales and clays^15,16^. Ilaro Formation is overlain by Coastal Plain Sands (Pliocene) and Recent Alluvium (Quaternary age) of Benin Formation which is youngest. The formation consists of sandstones and shales of upper Ilaro Formation, sequence of predominant continental sands and some lenses of shales and clays which is about 107.7 m thick.

## Materials and Methods

### Sample Collection and Preparation

**Figure 2:**
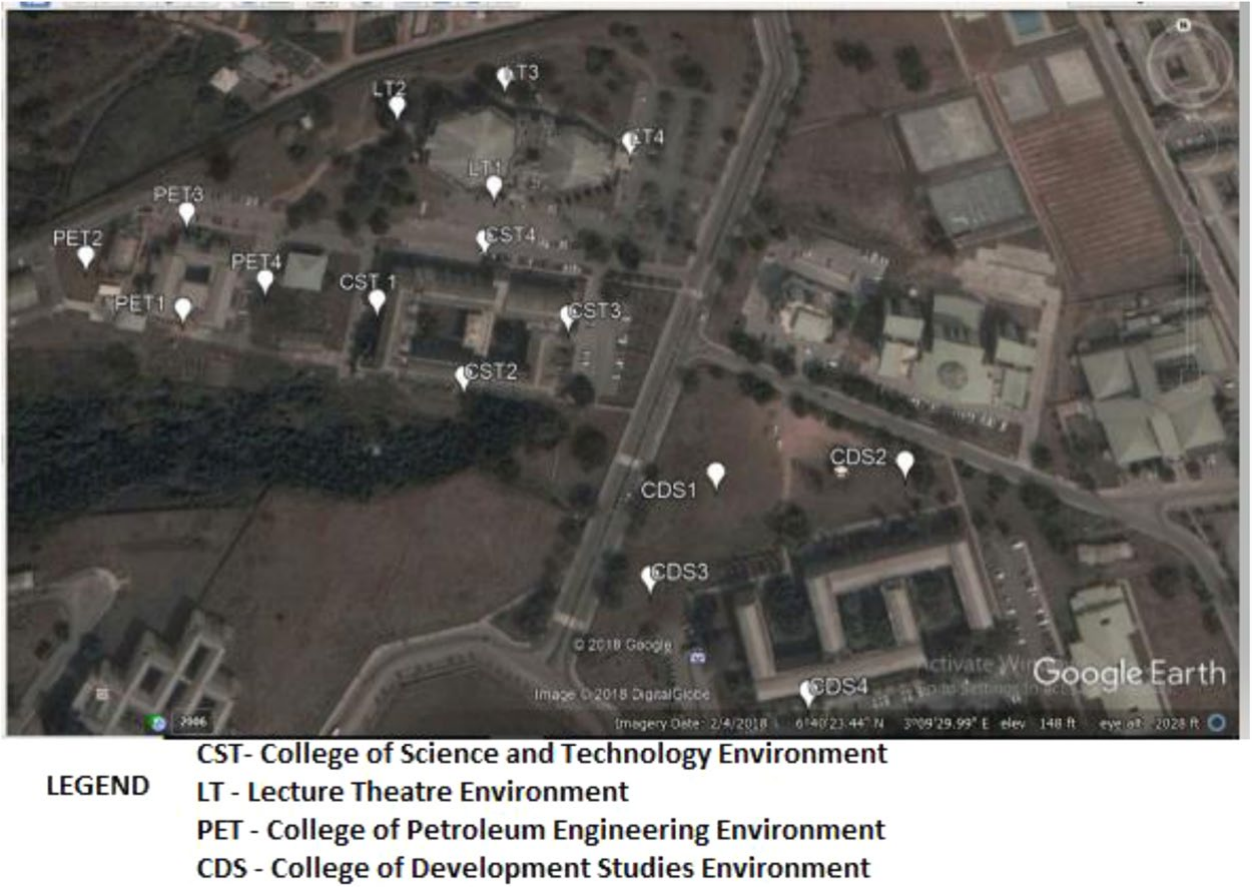
Image© 2018 DigitalGlobe.

Soil samples were collected around College of Science and Technology (CST) environment, Lecture theatre environment (LT), College of Petroleum Engineering environment (PET) and College of Development Studies (CDS) environment (Fig. 2) within the University community situated in coastaline area. Four samples were collected from each location thereby making the total number of sixteen soil samples. The sixteen (16) number of soil samples were collected at depth of 0 to 15 cm^17–19^ below ground level in and around Ado-Odo/Ota area. The samples were first kept under the ambient temperature between 28 and 30 °C for a week before drying with oven at about 105 °C for easy pulverization. Each soil sample was pulverized, passed through a sieve of 250 μm sieve size for homogeneity in powdered form. 1 kg of each sieved sample was weighed out and put in the polythene nylon and labeled accordingly for easy identification with permanent marker. Each soil sample crushed, pulverized and sieved which was stored in the polyethylene nylon bag was transferred to high density polyethylene bottle with a well labeled sample code using indelible marker^20^. Each bottle before filling with the sample was washed with borehole water 6 times and rinsing with distilled water (demineralized water). Thereafter, the samples were shipped to the Activation analysis Lab in Canada for gamma spectroscopy analysis. The organization that is responsible for the Lab is Activation Laboratories LTD or ACTLABS LTD and is located at 41 Bittern Street, Ancaster, Ontario Canada L9G 4V5. The Telephone number, FAX number, e-mail address and website are as follows: Telephone number: +1.905.648.9611 or +1.888.228.5227; FAX: +1.905.648.9613; E-MAIL: ancaster@actlabs.com and ACTLABS GROUP website: http://www.actlabs.com.

### Gamma Spectroscopic analysis of the Selected Soil Samples

The powdered soil samples in Activation Analysis Laboratory were weighed in 2 Oz seamless tin canisters (2 1/6″ diameter × 1 3/8″ height) canisters and sealed with an electrical tape for four weeks to achieve secular equilibrium between parents’ uranium and thorium with their daughter radionuclides. The high-resolution Gamma spectroscopy spectrums are acquired after 10000 seconds count. The Ra-226 (U-238) and Th-232 are determined from their progenies (Pb-214& Bi-214 for Ra-226 and Pb-212& Ac-228 for Th-232)^21^. The gamma characteristic and efficiency used in the determination of Uranium-228, Thorium-232 and Potassium-40 is shown in Table 1.

**Table 1:** Gamma characteristic and efficiency used for the determination of U-238, 232Th and 40 K.

Radioactive Series	Decaying Isotopes	Gamma-ray Energy (keV)	Percentage of Gamma Disintegration (%)
Uranium-238	^214^Pb (for ^238^U decay series) ^214^Bi (for ^238^U decay series)	352.0 609.4	35 43
Thorium-232	^208^Tl (for ^232^Th decay series) ^228^Ac (for ^232^Th decay series)	583.1 911.1	30 29
Potassium-40	^40^K	1460.8	10.68

### The Quality Assurance Control (QAC) and Calibration standards as well as the reference materials

One Blank, Standard and a Duplicate are included in each batch of ≤20 samples, to check the performance of the procedure. The Certified Reference Materials are also includes in a bath of samples. Calibration verifications are performed regularly. Method Detection Limit (MDL) is established analyzing 7 or more blanks taken through the analytical procedure. The average value, X, and the standard deviation of the values (S) is calculated. The MDL is equal to 3S (3 × standard deviation). A 24 hour count of the vacant shield should produce no activity for the nuclides of interest.

### Calibration and Standardization

The Energy calibration is performed regularly. The Energy file is stored in the Canberra Genie^TM^2000 V3.2 Spectroscopy Software Calibration files. Isotopes, associated gamma-ray energy, half-lives, and activities information, are listed in the certificate of calibration of multi-nuclide standard source sheet by the manufacturer. Efficiency calibration was completed by the manufacturer (Canberra) which should remain stable for the life of the instrument. The lead shield minimizes accuracy errors and interferences from the laboratory setting. The Certified Reference Materials (DL-1a, UTS-2, UTS-4, DH-1, BL-41, Bl-5, STM-2, IAEA-447 and IAEA-327) are used to calibrate the Gamma Spectroscopy counting system.

### Estimated soil radiological parameters

#### Radium equivalent radiological factor

This is the common factor used in comparing the radionuclides present in any material and this has been adopted in this present study for the purpose of comparing the measured radioactive concentration in the soil samples used. Radium equivalent activities were determined based on the estimation of 370 Bqkg^−1^ of Uranium-238, 259 Bqkg^−1^ of Thorium-232 and 4810 Bqkg^−1^ of Potassium-40 respectively. Each of this radionuclide produce gamma dose rate^22,23^ and Eq. (1) ^22,23^ was used in estimating the radium equivalent activity of the samples.

1 $${\rm{Raeq}}={{\rm{C}}}_{{\rm{Ra}}}+{\rm{1}}{{\rm{.43C}}}_{{\rm{Th}}}+{\rm{0}}{{\rm{.077C}}}_{{\rm{k}}}$$

#### Estimation of gamma dose rate of coastal area soil

The gamma dose rate for the soil samples used were estimated using Eq. (2) as applied by^22^ and^6^ respectively

2 $$\,{D}_{n}=0.462{C}_{Ra}+0.604{{\rm{C}}}_{{\rm{Th}}}+0.0417{{\rm{C}}}_{{\rm{K}}}$$

Further estimation was done in terms of the possible impact of these gamma dose rates emanating from study area where the soil samples were collected. This was estimated for both indoor (D_in_(nGyh^−1^)) and outdoor (D_out_(nGyh^−1^)) gamma dose rate using Eqs (3) ^6^ and (4) ^24^ respectively.

3 $${D}_{out}=0.427{C}_{Ra}+0.662{{\rm{C}}}_{{\rm{Th}}}+0.0432{{\rm{C}}}_{{\rm{K}}}$$

4 $${D}_{in}=1.4{{\rm{D}}}_{{\rm{out}}}$$

#### Annual effective dose rate equivalent (AEDR)

The indoors annual effective dose equivalent received by human is estimated from the indoor internal dose rate (Din), occupancy factor which is defined as the level of human occupancy in an area in proximity with radiation source; is given as 80% of 8760 hours in a year, and the conversion factor of 0.7 Sv Gy^−1^ which is used to convert the absorbed does in air to effective dose^6,23^. The annual effective dose equivalent for the sample used was evaluated using Eq. (5) ^6^.

5 $${\rm{AEDR}}=(0{\rm{.49}}\,{{\rm{C}}}_{{\rm{Ra}}}+{\rm{0}}{{\rm{.76C}}}_{{\rm{Th}}}+{\rm{0}}{{\rm{.048C}}}_{{\rm{K}}})\times {\rm{8.76}}\times {{\rm{10}}}^{-3}$$

#### External risk assessment (H_ex_)

The estimation of external risk assessment (H_ex_) associated to gamma dose rays emanating from the soil sample were determined using equation (6) as applied by^25^ and used by^23^.

6 $${{\rm{H}}}_{{\rm{ex}}}={{\rm{C}}}_{{\rm{Ur}}}/\mathrm{370}+{{\rm{C}}}_{{\rm{Th}}}/\mathrm{259}+{{\rm{C}}}_{{\rm{K}}}/\mathrm{4810}$$

C_Ur,_ C_Th_ and C_K_ are the concentrations of activities in B/kg.

#### Alpha index representative (Iα)

Alpha index representative (Iα) is one of the important radiological risk that have been developed in order to ascertain the safety of environment as a result of excess exposure to radiation emanating from the subsurface which use soil as a means of movement. Equation (7) has been used for the estimation^23,26,27^.

7 $$I{\rm{\alpha }}={{\rm{C}}}_{{\rm{Ur}}}/\mathrm{200}\,({{\rm{Bqkg}}}^{-{\rm{1}}})$$

#### Gamma representative index (Iγ)

Gamma representative index is usually used in the estimation of the **Iγ** hazard which is related to occurring natural radionuclides in any particular materials under study. The gamma index representation (Iγ) is estimated using Eq. (8) as used in^23,28,29^.

8 $$I{\rm{\gamma }}={{\rm{C}}}_{{\rm{U}}}r/\mathrm{300}({{\rm{Bqkg}}}^{-1})+{{\rm{C}}}_{{\rm{Th}}}/\mathrm{200}({{\rm{Bqkg}}}^{-1})+{{\rm{C}}}_{{\rm{K}}}/\mathrm{3000}({{\rm{Bqkg}}}^{-1})$$

#### Activity utilization index (AUI)

The use of the utilization activity index (UAI) for soil can be estimated through the sum of the radionuclides such as ^238^U^232^,Th and ^40^K and this can be estimated by the use of Eq. (9) as proposed by^30,31^

9 $$AUI=({C}_{Ur}/50Bqk{g}^{-1})fUr+({C}_{Th}/50Bqk{g}^{-1})fTh+({C}_{K}/500Bqk{g}^{-1})fk$$

where C_Th_, ${C}_{{Ur}}$, and C_K_ are the true figures of the activity in a unit of mass (Bqkg^−1^) of ^232^Th^238^,U, and ^40^K independently as analyzed in the materials such as soil. f_Ra_, f_Th_ and f_K_ are the contributions of the sum of dose rate at fractional level and is associated to gamma emission from the concentration of the actual activities of the studied radioactive materials.

## Result and Discussions

### Natural radioactivity concentration in coastal area soil

The concentration of natural-radioactivity measured in the sedimentary soil samples used in this investigation is presented in Table 2. The essence of this measurement is to ascertain the presence of this radioactive material in coastal area of Ado-Odo/Ota which defines the geological description of the study area. The result showed that U-238 ranged between 24 ± 7 and 49 ± 10 Bqkg^−1^and the mean values of uranium in the soil samples used was estimated to be 40.44 ± 9.13 Bqkg^−1^. The highest value of uranium was observed in the CST 3 sample while the lowest is observed in the soil sample PET 2. It was noticed that high values of uranium were observed in soil samples LT 1, LT 2, LT 3 and LT 4 with the indicated values of 45 ± 10, 43 ± 10, 47 ± 9 and 47 ± 10 Bqkg^−1^ respectively. The measured values of Th-232 in Bqkg^−1^ are shown in Table 1. The highest value of 120 ± 9 Bqkg^−1^ was noted in soil sample CST 1 while the lowest was noticed in soil sample CST 4 with the measured value of 67 ± 6 Bqkg^−1^. Higher values above 100 Bqkg^−1^ were observed in the soil samples CST 2, CST 3, PET 1 and CDS 2 respectively, however, the mean value for Th-232 were estimated to be 94.44 ± 7.38 Bqkg^−1^. Generally, high values of K-40 were observed in the soil samples used for this study. This observed high content of K-40 in the soil samples may be as result of geological formation underlying the study area. The mean value of K-40 estimated was low when compared with the similar work carried out by^17,18^ in Malaysia. However, the estimated mean value for K-40 were observed to be above 100 Bqkg^−1^.

**Table 2:** Measured radioactivity concentration in coastaline area soil of Ado-Odo/Ota.

Sample code	U-238 (Bqkg^−1^)	Th-232 (Bqkg^−1^)	K-40 (Bqkg^−1^)
CST 1	46 ± 10	120 ± 9	88 ± 17
CST 2	46 ± 10	106 ± 8	113 ± 17
CST 3	49 ± 10	118 ± 8	171 ± 18
CST 4	29 ± 8	67 ± 6	100 ± 14
LT 1	45 ± 10	94 ± 7	131 ± 17
LT 2	43 ± 10	97 ± 7	123 ± 17
LT 3	47 ± 9	99 ± 7	198 ± 19
LT 4	47 ± 10	99 ± 7	139 ± 20
PET 1	36 ± 10	119 ± 9	141 ± 19
PET 2	24 ± 7	86 ± 7	105 ± 17
PET 3	39 ± 8	83 ± 6	121 ± 17
PET 4	39 ± 7	69 ± 6	135 ± 17
CDS 1	37 ± 10	88 ± 8	145 ± 18
CDS 2	41 ± 8	103 ± 8	113 ± 15
CDS 3	40 ± 10	76 ± 7	179 ± 19
CDS 4	39 ± 9	87 ± 8	146 ± 18
**mean value**	**40.44 ± 9.13**	**94.44 ± 7.38**	**134.25 ± 17.44**

### Estimated Radium Equivalent

The radium equivalent factor was estimated using Eq. (1) ^21,23^ and the evaluated values are shown in Table 3. The estimated radium equivalent for the samples used ranged from 132.51 to 230.91 Bqkg^−1^ with mean value of 185.82 Bqkg^−1^. The observed value of Raeq in all the sample show that soil samples labeled with code CST 4 have the lowest value of Raeq while the highest is noted in soil sample CST 3. The other higher values above 200 Bqkg^−1^ were observed in soils samples CST 1, CST 2 and LT 3 respectively. In comparing the estimated mean of value of 185.82 Bqkg^−1^ with international reference limit of 370 Bqkg^−1^, it was inferred that mean value determined is within the recommended value as established or reported by^21^ and^6^.

**Table 3:** Radium equivalent, gamma dose rate of coastaline area soil.

Sample code	Ra_eq_(Bqkg^−1^)	D(nGyh^−1^)	D_in_(nGyh^−1^)	D_out_(nGyh^−1^)
CST 1	224.38	97.40	144.04	102.88
CST 2	206.28	89.99	132.57	94.70
CST 3	230.91	101.04	148.99	106.43
CST 4	132.51	58.04	85.48	61.06
LT 1	189.51	83.03	121.94	87.10
LT 2	191.18	83.58	123.05	87.89
LT 3	203.82	89.77	131.83	94.16
LT 4	199.27	87.31	128.27	91.62
PET 1	217.03	94.39	140.34	100.24
PET 2	155.07	67.41	100.40	71.72
PET 3	167.01	73.19	107.56	76.83
PET 4	148.07	65.32	95.43	68.16
CDS 1	174.01	76.29	112.45	80.32
CDS 2	196.99	85.87	126.81	90.58
CDS 3	162.46	71.85	105. 18	75.13
CDS 4	174.65	76.65	112.78	80.55
**mean value**	**185.82**	**81.32**	**119.82**	**80.65**

### Estimated Gamma dose rate (D_n_)

Gamma dose rate (Dn) was estimated using Eq. (2) ^6,21^. The estimated values ranged between 58.04 and 101. 04 nGyh^−1^ (Table 3). The observed result showed that sample CST 4 have the lowest value while the highest value of gamma dose rate was noted in soil sample CST 3. It was observed further that most soil samples have values of gamma dose rate that are higher than recommended limit value of 80 nGyh^−1^ except CST 4, PET 2, PET 3, PET 4, CDS 1, CDS 3 and CDS 4. In comparing the mean value with international accepted value, it was noted that the gamma dose rate exceeded the limit value of 80 nGyh^−1^. The estimation of outdoor and indoor gamma dose rate was done using Eqs 3 and 4. The estimated possible indoor and outdoor gamma dose rates were shown in Table 3. The estimation for the indoor gamma dose rate ranged from 85.48 to 148.99 nGyh^−1^ with mean value of 119.82 nGyh^−1^ while the outdoor gamma dose rate ranged between 61.06 and 106.43 nGyh^−1^ and the mean values was found to be 80.65 nGyh^−1^. The calculated mean of indoor gamma dose rate (D_in_) was compared with international reference value of 80 nGyh^−1^ ^6^, the observation showed that the indoor gamma dose rate of the soil samples used is almost 2 times higher than recommend limit^6^. Furthermore, estimated mean of outdoor gamma dose rate (D_out_) was compared with international reference value of 50 nGyh^−1^ as reported by^24^, it was noticed that the estimated values were higher, which may be as a result of geological formations (such as gypsum, kaolin) which predominantly underlying the study area. This also suggests that there may be possible health issues associated with the soil of the study area.

### Annual effective dose rate equivalent (AEDR)

The annual effective dose rate equivalent for the sample used in this study was evaluated using Eq. (5). The estimated values of AEDR ranged from 0.61 to 1.07 mSv y^−1^. The mean value was evaluated to be 0.86 mSv y^−1^. The higher values of AEDR above 0.07 mSv y^−1^ ^6^ were noticed in all the soil samples. In comparing the mean value of AEDR with reference value 0.07 mSv y^−1^ ^6^, it was observed that the estimated value was higher than recommended value of 0.07 mSv y^−1^ as established by^6^.

### Estimation of External risk assessment (H_ex_)

The estimation of external risk assessment (H_ex_) associated to gamma dose rays emanating from the soil sample were determined using Eq. (6) ^25,23^. The values estimated for external risk in the soil samples are shown in Table 4. The evaluated external risk ranged between 0.36 and 0.62. The highest value of H_ex_ (0.62) is noted in soil sample CST 3 and the lowest value observed in soil sample CST 4 (0.36). The mean value of external risk was estimated to be 0.50. The acceptable limit for hazard in soil is unity or 1, in this present study, the estimated mean value and the external risk value for each samples were within the standard value of unity as recommended by^6^.

**Table 4:** AEDR, H_ex_, I_α_, I_γ_, and AUI of coastaline area soil formation.

Sample code	AEDR(mSv y^−1^)	Hex	Iα (Bq/kg)	Iγ(Bq/kg)	AUI
CST 1	1.03	0.61	0.23	0.78	1.88
CST 2	0.95	0.56	0.23	0.72	1.72
CST 3	1.07	0.62	0.25	0.81	1.89
CST 4	0.61	0.36	0.15	0.47	1.09
LT 1	0.87	0.51	0.23	0.66	1.56
LT 2	0.88	0.52	0.22	0.67	1.58
LT 3	0.94	0.55	0.24	0.72	1.65
LT 4	0.92	0.54	0.24	0.70	1.64
PET 1	1.01	0.59	0.18	0.76	1.78
PET 2	0.72	0.42	0.12	0.55	1.27
PET 3	0.77	0.45	0.20	0.59	1.37
PET 4	0.68	0.40	0.20	0.52	1.21
CDS 1	0.81	0.47	0.19	0.61	1.42
CDS 2	0.91	0.53	0.21	0.69	1.63
CDS 3	0.75	0.44	0.20	0.57	1.30
CDS 4	0.81	0.47	0.20	0.61	1.42
**mean value**	**0.86**	**0.50**	**0.21**	**0.65**	**1.53**

### Alpha index representative (Iα) Estimation

Alpha index representative (Iα) is one of the important radiological risk that have been developed in order to ascertain the safety of environment, Eq. (7) has been used for the estimation [25; 26; 22]. The estimation of Iα ranged from 0.12 to 0.25 with mean value of 0.21 and is shown in Table 3. The estimated H_ex_ values for all the samples used were below the recommended value of unity or 1^6^.

### Gamma representative index (Iγ) Estimation

In this present study, Eq. (8) has been used^28^. The gamma representative index is used mostly in the identification of criterion controls of dose range of 0.3 to 1 mSvy^−1^ ^29^. The gamma representative index estimated is shown in Table 3. The result showed that soil sample CST 4 has the lowest value of 0.47 while soil sample CST 3 has the highest value 0.81. Further observation showed all the soil samples used in this study have high values above minimum limit of 0.3 as recommended by^29^, however, the gamma representative index estimation in the soil samples were below the maximum limit of 1 mSvy^−1^.

### Estimated Activity utilization index (AUI)

The activity utilization index (AUI) for soil samples used in this study have been estimated using Eq. (9) as proposed by^30,31^. In order to ascertain the contribution of activity utilization index in soil, the estimated value should be less than 2 and is equivalent to a dose rate of 80 nGyh^−1^ ^30^. The utilization index ranged between 1.09 and 1.89 with mean value of 1.53. Estimated AUI for each soil samples and the mean value were observed to be less 2 than as recommended by^30^. However some soil samples having values close to 2 were observed in some soil samples such as CST 1, CST 2, CST 3 and PET 1 respectively.

## Conclusions

Investigation of natural environmental radioactivity in coastaline area soil of Ado-Odo/Ota has been carried out to ascertain the existence or presence of radionuclides (U-238, Th-232 and K-40) in the study area. Gamma-ray spectroscopy (HPGe detector) was used for the measurement of radioactive concentration present in the sixteen (16) soil samples used. The estimated radium equivalent for the samples used ranged from 132.51 to 230.91 Bqkg^−1^ with mean value of 185.89 Bqkg^−1^. The mean value of 81.32 nGyh^−1^ for the gamma dose rate of the soil samples exceeded the limit value of 80 nGyh^−1^. The estimation for the indoor gamma dose rate ranged from 85.48 to 148.99 nGyh^−1^ with mean value of 119.82 nGyh^−1^ while the outdoor gamma dose rate ranged between 61.06 and 106.43 nGyh^−1^ and the mean values was found to be 80.65 nGyh^−1^. The estimated values of annual effective dose equivalent (AEDRE) ranged from 0.61 to 1.07 mSv y^−1^. The mean value of external risk was estimated to be 0.50. The estimation of alpha index representative (Iα) ranged from 0.12 to 0.25 with mean value of 0.21 while the gamma representative index ranged between 0.47 and 0.81. The activity utilization index (AUI) ranged from 1.09 to 1.89 with mean value of 1.53. In comparing the mean values of radiological parameters estimated with standard value as suggested by^6^, it was observed that all the estimated values of radiological parameters were within the range of international reference value except the gamma dose rate and annual effective dose equivalent which may be as a result of geological formation of the study area which is predominantly dominated by kaolin and gypsum.

## Supplementary information

Calculated value from radioactive concentration

## Data Availability

The data used for this study would be made available upon request.
