# Supplementary material for: Investigation of natural environmental radioactivity concentration in soil of coastaline area of Ado-Odo/Ota Nigeria and its radiological implications
Source: Sci Rep. 2019 Mar 12;9:4219. doi: 10.1038/s41598-019-40884-0 (PMC6414599; doi:10.1038/s41598-019-40884-0)
Supplement: Supplementary file 1 — Calculated value from radioactive concentration [file 41598_2019_40884_MOESM1_ESM.pdf]

Investigation of natural environmental radioactivity concentration in soil of coastaline area of Ado-Odo/Ota Nigeria and its radiological implications

[1*]E.S. Joel, [1]O. Maxwell, [1]O.O Adewoyin, [1]O.C Olawole, [1]T.E. Arijaje, [2]Z. Embong [3]M.A Saeed

Estimated value of radiological parameters

sample code	Raeq(Bqkg-1)	D(nGh-1)	Din(mSvy-1)	Dout(mSvy-1)
CST 1	224.376	97.4016	0.472904248	0.118226062
CST 2	206.281	89.9881	0.344045784	0.086011446
CST 3	230.907	101.0407	0.179136974	0.044784243
CST 4	132.51	58.036	0.281776387	0.070444097
LT 1	189.507	83.0287	0.403120944	0.100780236
LT 2	191.181	83.5831	0.405812667	0.101453167
LT 3	203.816	89.7666	0.435834796	0.108958699
LT 4	199.273	87.3063	0.423889548	0.105972387
PET 1	217.027	94.3877	0.458271161	0.11456779
PET 2	155.065	67.4105	0.32729146	0.081822865
PET 3	167.007	73.1957	0.355379763	0.088844941
PET 4	148.065	65.3235	0.317158657	0.079289664
CDS 1	174.005	76.2925	0.370415346	0.092603837
CDS 2	196.991	85.8661	0.416897089	0.104224272
CDS 3	162.463	71.8483	0.348837866	0.087203398
CDS 4	174.652	76.6542	0.372171472	0.093042868
**mean value**	**185.820375**	**81.3206**	**0.36955901**	**0.092389373**

sample code	AEDR(mSv y−1)	Hex	Iα (Bq/kg)	Iγ(Bq/kg)	AUI
CST 1	1.03336464	0.60594	0.23	0.782667	1.881856
CST 2	0.93858144	0.557083	0.23	0.721	1.714786
CST 3	1.06782648	0.623582	0.245	0.810333	1.892222
CST 4	0.6125868	0.357856	0.145	0.465	1.08552
LT 1	0.87405528	0.484556	0.225	0.663667	1.562062
LT 2	0.93248448	0.516305	0.215	0.669333	1.579166
LT 3	0.94410024	0.550431	0.235	0.717667	1.646436
LT 4	0.91929192	0.538165	0.235	0.698	1.641598
PET 1	1.00606848	0.586071	0.18	0.762	1.781722
PET 2	0.7197216	0.418741	0.12	0.545	1.26925
PET 3	0.77086248	0.451025	0.195	0.585333	1.372922
PET 4	0.6835428	0.399881	0.195	0.52	1.20495
CDS 1	0.8056572	0.469914	0.185	0.611667	1.41681
CDS 2	0.90923544	0.531987	0.205	0.689333	1.632346
CDS 3	0.75293952	0.438759	0.2	0.573	1.302358
CDS 4	0.80884584	0.471666	0.195	0.613667	1.423292
**mean value**	**0.86119779**	**0.500123**	**0.2021875**	**0.651729**	**1.525456**
